# Different expression of circulating microRNA profile and plasma SP-D in Tibetan COPD patients

**DOI:** 10.1038/s41598-022-05592-2

**Published:** 2022-03-01

**Authors:** Xue-feng Shi, Xiang He, Ze-rui Sun, Jian-xiang Wang, Yu-hai Gu, You-bang Xie, Jie Duo

**Affiliations:** 1 Department of Respiratory Medicine, Qinghai Provincial People’s Hospital, Xining, Qinghai 810007 People’s Republic of China; 2 Department of Hematology and Rheumatology, Qinghai Provincial People’s Hospital, Xining, Qinghai 810007 People’s Republic of China

**Keywords:** Gene expression profiling, Diagnosis, Epigenomics

## Abstract

COPD is the fourth leading cause of mortality, and is predicted to be the third leading cause of death worldwide by 2020. But few studies on Tibetan COPD of China. This study identifies distinctive miRNA signatures in Tibetan COPD patients from Tibetan healthy subjects that could serve as diagnostic biomarkers or describe differential molecular mechanisms with potential therapeutic implications. In this study, a total of 210 differentially expressed miRNAs were screened. Analysis of the functions of target genes of differentially expressed miRNAs via GO enrichment *analysis* revealed that they mainly influenced guanyl-nucleotide exchange factor activity, cell morphogenesis and the positive regulation of GTPase activity. KEGG pathway enrichment analysis showed that these target genes were mainly enriched in signaling by NGF, Axon guidance, developmental biology, ubiquitin mediated proteolysis, and PDGF signaling pathways. MiR-106-5p and miR-486-5p expression was validated in the complete cohort. Age, plasma miR-106-5p, miR-486-5p, SP-D protein levels, and SP-D mRNA level were also determined to be correlated with FEV1%Pred, and may as the risk factors of Tibetan COPD. The combination of plasma miR-106-5p, miR-486-5p and SP-D mRNA expression may be the best model to assist the diagnosis of Tibetan COPD.

## Introduction

Chronic obstructive pulmonary disease (COPD) is an incompletely reversible, preventable, and treatable disease with airflow limitation characterized by high morbidity and mortality worldwide. It is estimated that more than 3 million people die each year from COPD, accounting for an estimated 6% of total deaths globally. COPD is often associated with comorbidities^1^, such as chronic pulmonary heart disease and respiratory failure.

MicroRNAs (miRNAs) are a class of post-transcriptional regulators that have been found to have a promoting role in lung development, maturation, and the maintenance of lung function^2,3^. Dysregulated miRNA expression might be a direct consequence of an indirect effect of airway disease onset or progression. In recent years, relevant studies have demonstrated that miRNAs are involved in the pathogenesis of most human diseases, and some studies have demonstrated that miRNAs are involved in the physiopathological mechanisms of a variety of respiratory diseases^2–4^, indicating the importance of miRNAs in the pathogenesis of respiratory diseases, including COPD. The complicated interaction between genetics, protein synthesis, and immune response in COPD is even more intricate when miRNAs regulation is introduced. These small noncoding RNAs are implicated in the immune response of COPD^5^. They act by negatively regulating the expression of key immune development genes, thus contributing important logic elements to the regulatory circuitry.

miRNAs have first been as biomarkers for cancer in 2008^6^, and ever since, more and more literature mentioned them as biomarkers for numerous diseases^7^. Plasma miRNAs are relatively stable, easily accessible, and can be measured in a non-invasive way, which suggests their potential as ideal biomarkers for diagnosis and prediction of disease progression in a variety of afflictions. Otherwise, miRNAs also can be used as multimarker models for diseases diagnosis, treatment guidance and the evaluation of treatment responsiveness^7,8^. It has already been reported that differentially expressed miRNAs between healthy and COPD patients participated in organelle fission, inflammatory processes, and airway remodeling of COPD^9,10^. Several studies also showed that SP-D are correlated with severity of COPD and might be valuable indicators of lung injury^11,12^. But few studies on Tibetan of China. So we determined to study the differential expressed miRNAs and SP-D expression in the process of COPD in Tibetan populations of China.

## Subjects and methods

### Study patients characteristics

**Figure 1:**
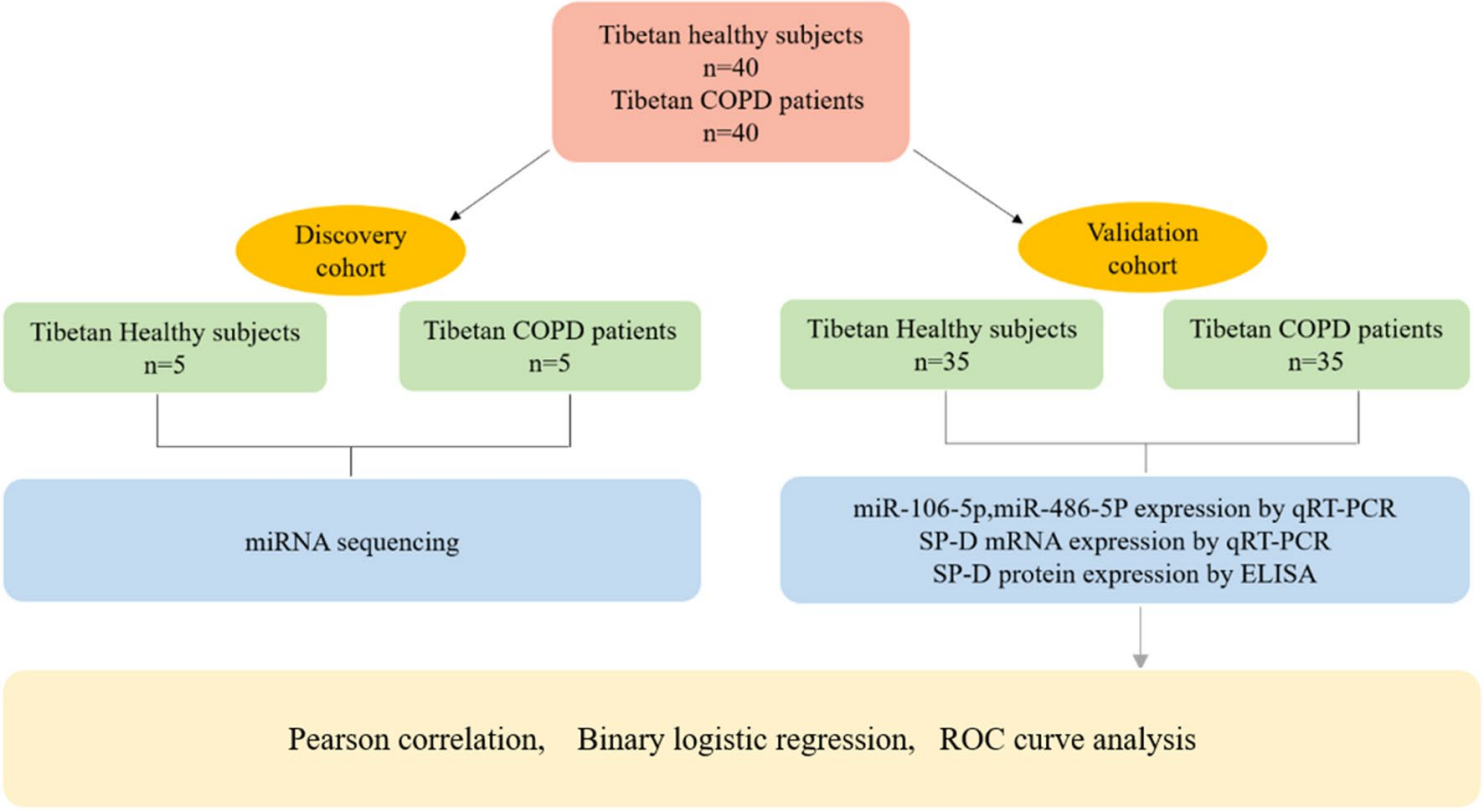
Study scheme.

The present study was approved by the Ethics Committee of Qinghai Provincial People's Hospital (Approval NO. 2018-53 and 2018-54), and performed in accordance with relevant guidelines/regulations and the Declaration of Helsinki. The patients of this study and/or their guardians were informed and signed an informed consent form. 40 Tibetan healthy subjects were selected as the control group, and 40 Tibetan COPD patients from January 2019 to January 2021 as COPD group, who signed an informed, written consent form, diagnosed with COPD (post-bronchodilator FEV1/FVC ≤ 70%). Of them, five cases from each group were choose for discovery cohort, and left 35 cases from each group were choose for validation cohort (Fig. 1). All COPD Patients meet the diagnostic criteria of GOLD2017, and exclude other diseases causing airflow limitation. Patients suffering from other respiratory diseases, or combined with endocrine, metabolic, allergic and autoimmune diseases, tumors and other serious systemic serious primary diseases were excluded from this study. Recruited patients underwent socio-demographic and clinical questionnaires, lung function tests and blood extraction. Plasma was isolated and frozen at − 80 °C.

*Discovery cohort and miRNA sequencing* Five Tibetan healthy subjects and five Tibetan COPD patients were selected for high-throughput sequencing of miRNAs. There were 3 males and 2 females enrolled in the control group, with an average age of 64.00 ± 3.54 years. There were 4 males and 1 female in the COPD group, with an average age of 76.00 ± 2.16. There was no statistical significance in gender, age and smoking history between two groups. Predicted FEV1% (FEV1%Pred) and FEV1/FVC(%) of COPD patients were lower than those of Tibetan healthy people. Details are showed in Table 1. Total plasma RNA was extracted with Trizol (Tiangen, Beijing) and assessed with Agilent 2100 BioAnalyzer (Agilent Technologies, Santa Clara, CA, USA) and Qubit Fluorometer (Invitrogen). Sequence libraries were generated and sequenced by CapitalBio Technology (Beijing, China). A total amount of 3ug total RNA per sample was used as input material for the small RNA library. Illumina Hiseq 2500 platform was used to sequence the library preparations and 50 bp single-end reads were generated.

**Table 1:** Characteristics of discovery cohort subjects.

	Control group	COPD group		*P*-value
Age (years)	64.00 ± 3.54	71.80 ± 9.58	t = 1.709	0.126
**Gender (cases/%)**			X^2^ = 0.476	0.490
Male	3 (40%)	4 (60%)		
Female	2 (60%)	1 (40%)		
**Smoking history (n/%)**			X^2^ = 0.400	0.527
Yes	2(40.00)	3(60.00)		
No	3(60.00)	2(40.00)		
FEV1% predicted (%)	85.22 ± 4.15	47.94 ± 11.56	t = 6.786	0.001
FEV1/FVC (%)	82.35 ± 5.34	52.68 ± 8.68	t = 6.511	0.000

*Validation cohort* Thirty-five samples from two groups were selected for validation of differentially expressed miRNAs. There were 24 males and 11 females enrolled in control group, with an average age of 66.68 ± 5.86 years. 25 males and 10 females in COPD group were enrolled in COPD group, with an average age of 66.89 ± 8.05.

### RNA extraction, miRNA reverse transcription and miRNA polymerase chain reaction (PCR)

Total RNA was extracted from plasma using TRIzol reagent (Ambion; Thermofisher Scientific, Inc.). Total RNA obtained from plasma was transcribed to cDNA using the TaqMan® MicroRNA Reverse Transcription kit (Applied Biosystems Life Technologies; Thermo Fisher Scientific, Inc.), and qRT-PCR amplification with TaqMan™ Universal MixII (Applied Biosystems Life Technologies; Thermo Fisher Scientific, Inc.). U6 was used as an internal control. All primers (U6, miR-486-5p, miR-106b-5p) corresponding to miRNAs were bought from Applied Biosystems (Thermo Fisher Scientific, Inc. Cat. No. 4427975, 4427975, 4427975). The expression of SP-D were detected by SYBR Green system and normalized with β-actin. The primers were as follows: SP-D, sense 5’-GGGAGAAGATTTTCAAGACAGC-3’ and antisense 5’-CCTCTGTCTTGGAATCAGTCAT-3’; β-actin, sense 5’-GCGGGAAATCGTGCGTGAC-3’ and antisense 5’-GGAAGGAAGGCTGGAAGAG -3’; qRT-PCR analysis was performed using an ABI Prism 7500 Sequence Detector (Applied Biosystems, FosterCity, CA, USA), and calibrated by using the 2^-ΔΔCT^ method.

*ELISA analysis* Plasma of Tibetan healthy people and COPD patients were subjected to ELISA analysis for their concentration of SP-D. SP-D ELISA kits from Bioswamp (Wuhan, Hubei, China) were used according to the manufacturer's instructions.

### Statistical analysis

All values are presented as the mean ± SD. SPSS 19.0 software was used for statistical analysis. After quantile normalization and quality control, statistical significance of the differentially expressed miRNAs was assessed by unpaired t-test using a *p*-value cut-off of 0.05 and a fold-change 2.0. miRNA expression levels were estimated by TPM (transcript per million): Normalization formula: Normalized expression = mapped readcount/Total reads * 1,000,000. Based on our discovery cohort results, we use PASS 15.0.5 to calculate the sample size of validation cohort (two independent means). Various variables were analyzed using Pearson correlation, and all included variables are normally distributed. Binary logistic regression models are used to study effects of predictor variables (Age, sex, smoking history, SP-D protein level, SP-D mRNA level, miR-106-5p, and miR-486-5p) on presence or absence of COPD, and forward stepwise regression of model building approach was chosen. The Hosmer–Lemeshow goodness-of-fit tests was used measure of model fit. ROC curve analysis, based on predicted probability values from binary logistic regression models, differential expressed miRNAs and SP-D level, was used to evaluate the diagnostic performance for Tibetan COPD. Differences between groups were significant at *P* < 0.05.

### Ethics approval and consent to participate

The study was approved by the Ethics Committee of Qinghai Provincial People's Hospital (Approval NO. 2018-53 and 2018-54), and performed in accordance with relevant guidelines/regulations and the Declaration of Helsinki. The patients of this study and/or their guardians were informed and signed an informed consent form.

## Results

### Patient characteristics

Thirty-five Tibetan patients with COPD and Thirty-five Tibetan healthy people were included in this study as validation study. Characterization of the demographic, clinical and functional features of the entire population are shown in Table 2. Briefly, there was no statistical significance in age, gender, and smoking history between two groups. Moreover, COPD patients showed significantly lower predicted FEV1%Pred and FEV1/FVC than control healthy people.

**Table 2:** Characteristics of validation cohort subjects.

	Control group	COPD group		*P*
Age (years)	66.89 ± 5.86	66.89 ± 8.05	t = 0.119	0.960
**Gender (ratio/%)**			X^2^ = 0.068	0.794
Male	24 (66.7%)	25 (63.3%)		
Female	11 (33.3%)	10 (36.7%)		
**Smoking history**			X^2^ = 0.516	0.473
Yes	18 (51.00)	15 (43.00)		
No	17 (49.00)	20 (57.00)		
Number of acute exacerbations	/	1.71 ± 0.85		
mMRC	/	2.63 ± 0.93		
FEV1% predicted (%)	80.59 ± 7.73	45.35 ± 7.70	t = 19.113	0.000
FEV1/FVC (%)	84.67 ± 7.68	48.00 ± 12.04	t = 15.185	0.000

### Difference in circulating miRNA expression profile of COPD in Tibetan population

**Figure 2:**
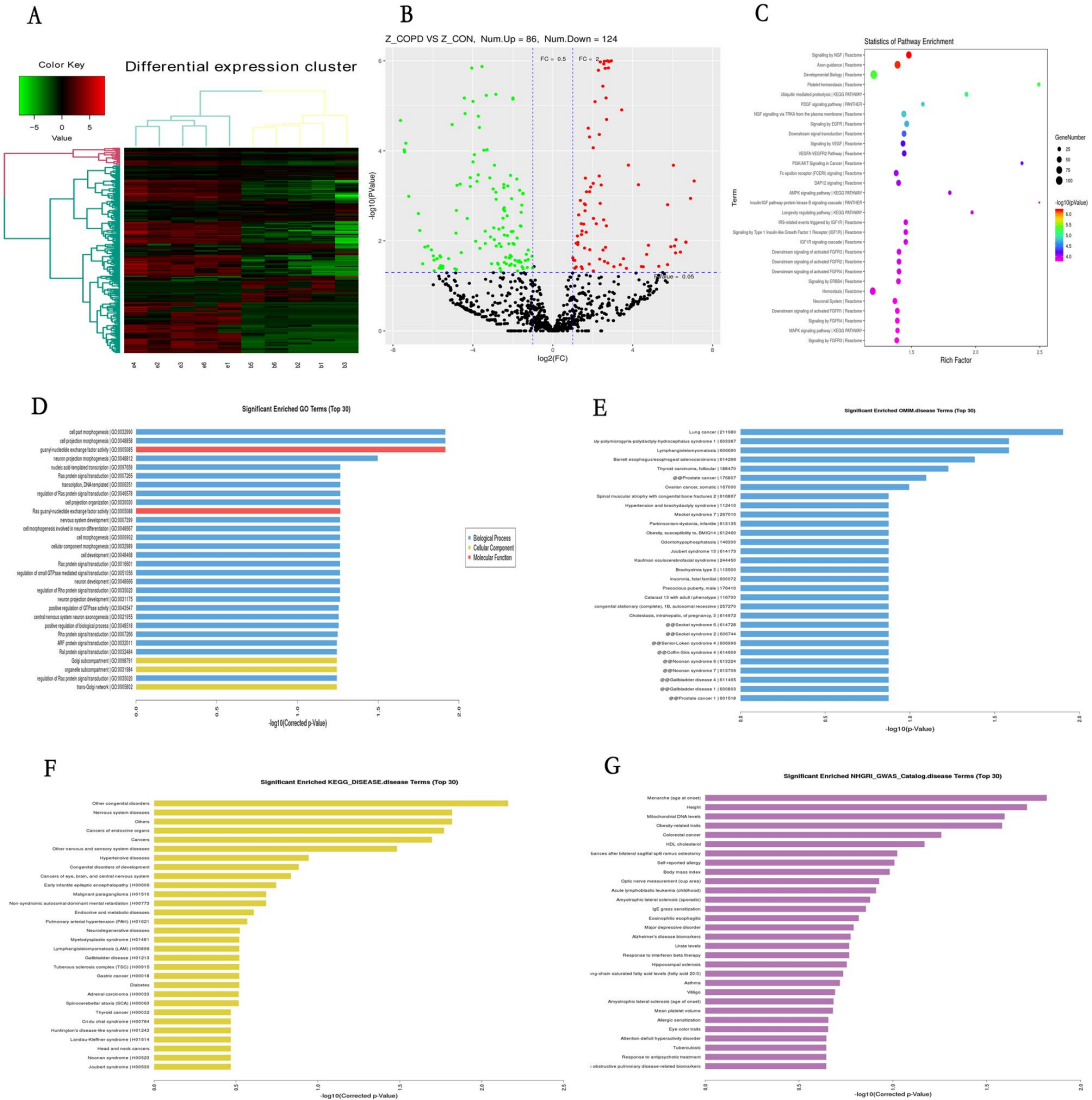
MiRNA expression profile Tibetan healthy people and Tibetan COPD patients by Illumina novaseq 6000. (**A**) Comparison of cluster data between Tibetan COPD patients and Tibetan healthy subjects. (**B**) Volcano plot of differential miRNAs of Tibetan COPD and healthy subjects. The green dots on the left of the graph show downregulated miRNAs with log2(Fold change, FC) ≤ 1, and the red dots on the right of graph show upregulated miRNAs with log2FC ≥ 1. (**C**) Predicted target gene of differentially expressed miRNAs pathway enrichment. (**D**) Enriched GO of predicted target gene-top30. (**E**) OMIM. diseases enrichment enrichment analyses. (**F**) KEGG diseases enrichment analyses-top30. (**G**) NHGRI GWAS Catalog enrichment analyses-top30.

A discovery set of samples was selected from the Tibetan control group and COPD group for high-throughput sequencing. Raw fastq reads were processed with bcl2fastq. The small RNA tags were mapped to reference sequence using Bowtie-1.1-1 without mismatch to analyze their expression and distribution on the reference genome. The heatmap of gene expression in both groups, obtained using the Cluster software, showed the difference in the expression of each gene in the two groups. In the diagram with x-axis of log2 (fold change, FC) and y-axis of -log10 (*P*-value), the data closer to the left and right bottom corresponded to the lower *P*-value, larger fold change, and more significant difference. A total of 210 differentially expressed miRNAs were screened by FC ≥ 2, and *P* value < 0.05. 124 miRNAs were downregulated, and 86 miRNAs were upregulated. Table 3 showed 34 downregulated miRNAs and 14 upregulated miRNAs screened by log2FC > 2 or < -3, and *p* value < 0.05. A heatmap of Cluster analysis was performed for the differential expressed miRNAs in 5 cases Tibetan healthy control group and 5 cases Tibetan COPD group (Fig. 2A) a. As showed in Fig. 2B, the data closer to the left and right bottom corresponded to the lower *P*-value, larger fold change, and more significant difference.

**Table 3:** MiRNA profiling of Tibetan-con vs Tibetan-COPD groups.

	Tibetan-con VS Tibetan-COPD	
	Log2 (FC)	*p*-value
hsa-miR-766-5p	− 7.6215362	2.01E-05
hsa-miR-452-5p	− 7.4168887	0.000105
hsa-miR-6810-5p	− 7.2086024	0.00102264
hsa-miR-889-3p	− 6.7247246	0.00248057
hsa-miR-3120-3p	− 6.6547623	0.00587197
hsa-miR-487b-5p	− 6.5119272	0.03232667
hsa-miR-433-3p	− 6.2468536	0.01308148
hsa-miR-543	− 5.9424356	0.02664891
hsa-miR-412-5p	− 5.9302398	0.04627339
hsa-miR-6763-5p	− 5.6813431	0.04030393
hsa-miR-556-3p	− 5.6477788	0.02143054
hsa-miR-1269b	− 5.6086283	0.02277529
hsa-miR-6715a-3p	− 5.5097197	0.04021535
hsa-miR-374b-3p	− 5.5015826	0.03644857
hsa-miR-548b-3p	− 5.2113904	0.02484112
hsa-miR-494-3p	− 5.0393846	2.51E-05
hsa-miR-32-3p	− 4.8993955	0.04177692
hsa-miR-376a-3p	− 4.8451073	0.00672373
hsa-miR-20a-3p	− 4.8219925	0.04330541
hsa-miR-551a	− 4.6345547	0.00054572
hsa-miR-548e-5p	− 4.5804855	0.0181971
hsa-miR-4286	− 4.3891673	1.39E-05
hsa-miR-6852-5p	− 3.9916538	0.0001749
hsa-miR-301b-3p	− 3.8597853	0.01022904
hsa-miR-1273 h-5p	− 3.7930114	0.00047547
hsa-miR-6721-5p	− 3.7294376	0.00034081
hsa-miR-654-3p	− 3.6720771	2.93E-05
hsa-miR-12135	− 3.6477998	0.01463811
hsa-miR-409-3p	− 3.5426919	3.38E-07
hsa-miR-330-3p	− 3.3797022	0.00609024
hsa-miR-6813-5p	− 3.3750543	0.01143905
hsa-miR-4433b-5p	− 3.2910686	9.37E-05
hsa-miR-6772-3p	− 3.118043	0.00819348
hsa-miR-301a-5p	− 3.0635331	0.03228616
hsa-miR-106b-5p	2.03551141	0.00317553
hsa-miR-1270	2.31395864	0.00020779
hsa-miR-183-5p	2.32379475	0.00063528
hsa-miR-16–2-3p	2.37192325	2.22E-08
hsa-miR-486-3p	2.42273034	1.60E-05
hsa-miR-20b-5p	2.50587691	0.04330541
hsa-miR-296-5p	2.5457103	0.04790614
hsa-miR-15b-5p	2.57825873	4.14E-08
hsa-miR-5010-5p	2.63855605	0.01391818
hsa-miR-486-5p	2.74539478	5.72E-06
hsa-miR-548 h-3p	3.37625357	0.033913
hsa-miR-548z	3.53659453	0.02488495
hsa-miR-629-3p	3.68391331	0.04076206
hsa-miR-548az-5p	3.87006793	0.01296323

### Predicted target genes of differentially expressed miRNAs

Target genes were predicted based on miRanda 3.3a by Score ≥ 140, and Energy ≤ − 20 kcal/mol. There were total 3934 target genes selected by top10 target genes of each miRNAs.

### Enrichment analysis of predicted target genes of differentially expressed miRNAs

Analysis of the functions of target genes of differentially expressed miRNAs via GO enrichment *analysis* revealed that they mainly influenced guanyl-nucleotide exchange factor activity, cell morphogenesis and the positive regulation of GTPase activity. Figure 2D. KEGG pathway enrichment analysis showed that these target genes were mainly enriched in signaling by NGF, Axon guidance, developmental biology, ubiquitin mediated proteolysis, and PDGF signaling pathways. Among them, developmental biology was enriched the most in target genes (Fig. 2C). Diseases enrichment was obtained by OMIM, KEGG, and NHGRI GWAS Catalog enrichment analyses. KEGG enrichment showed pulmonary arterial hypertension was the 14th disease, which is the main complication of COPD (Fig. 2F). OMIM enrichment showed lung cancer was the 1st disease which is consistent with that COPD patients at higher risk of developing lung cancer^13^ (Fig. 2E). COPD-related biomarkers was the 30th by NHGRI GWAS Catalog enrichment analyses (Fig. 2G).

### Plasma miRNA-106-5p, miRNA-486-5p, SP-D protein and SP-D mRNA expression between the COPD patients and control group

**Figure 3:**
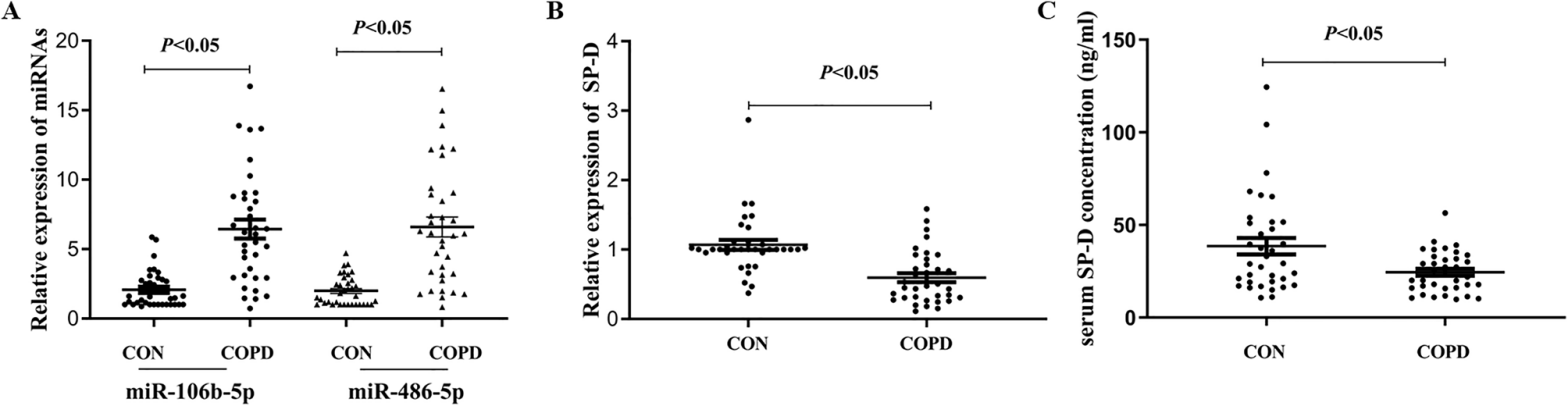
miR-486-5p, miR-106-5p and SP-D expression in Tibetan COPD patients and Tibetan healthy subjects. (**A**) Plsama miR-486-5p and miR-106-5p expression-qRT-PCR; (**B**) Plasma SP-D mRNA expression-qRT-PCR; (**C**) Plasma SP-D protein expression-Elisa.

As showed in Table 3, there were 14 upregulated miRNAs[Log2(FC) ≥ 2] between COPD patients and control group. Our previous study showed that miR-486-5p was a hypoxia related miRNA^14^, and COPD patients are in a hypoxia situation because of the lung function injury. At the same time, miR-106b-5p was reported acting as a potential marker in pulmonary arterial hypertension (PAH)^15^. And reccurrent exacerbations of COPD also lead to PAH. So we validatd plasma miRNA-106-5p and miRNA-486-5p expression in Tibetan COPD patients, utilizing an expanded sample size by qRT-PCR. As showed in Fig. 3A, miR-106b-5p and miR-486-5p expression were significantly higher in Tibetan COPD patients than Tibetan healthy people which is consistent with miRNAs profiling results. In addition, we also measured the expression levels of SP-D, and showed that plasma SP-D mRNA and protein expression all decreased in Tibetan COPD group compared with the control group (Fig. 3B, C).

### The correlation analysis of Tibetan COPD severity

**Figure 4:**
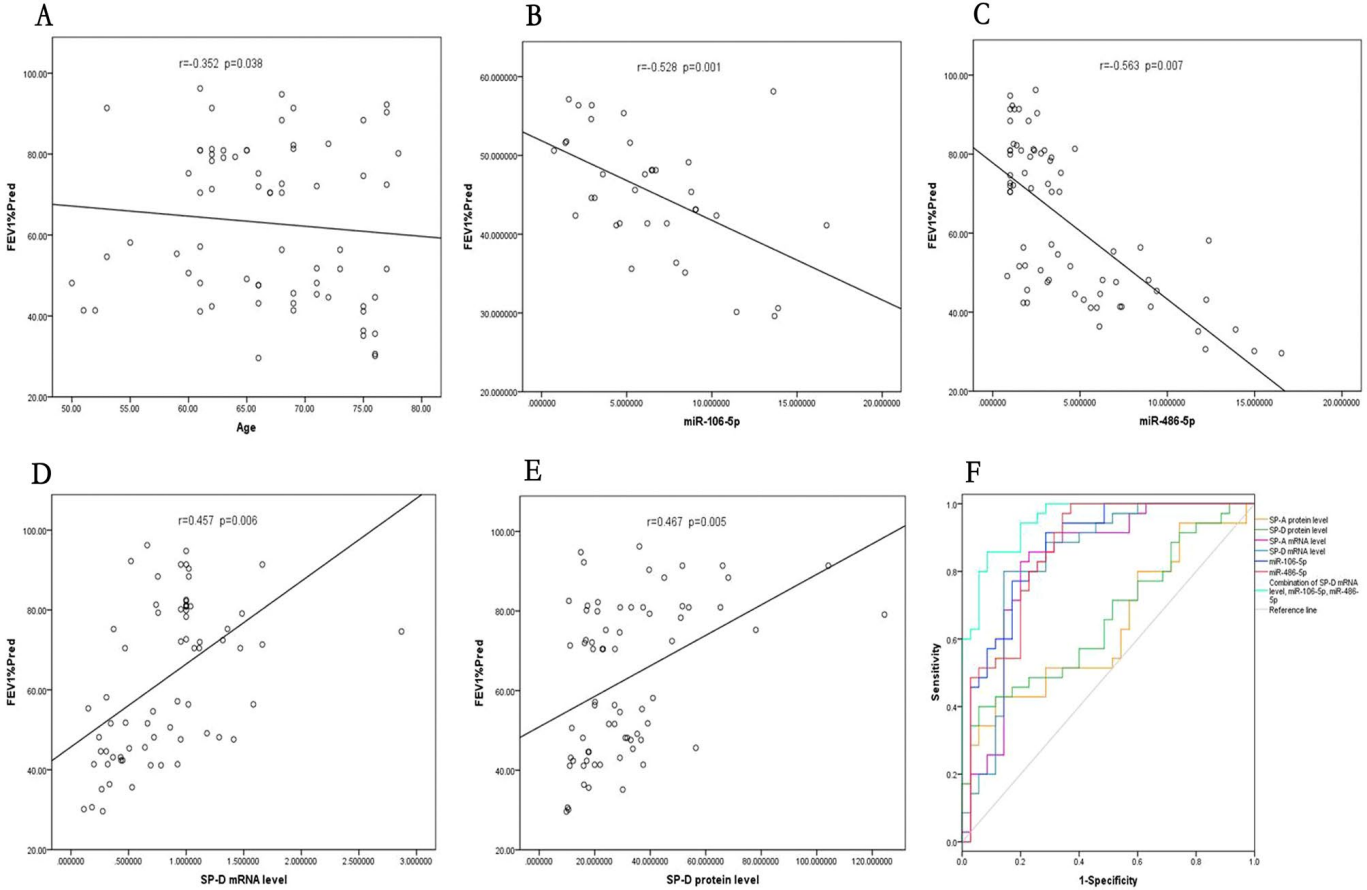
(**A**–**E**) The correlations of age, miR-486-5p, miR-106-5p and SP-D expression with FEV1%Pred in Tibetan COPD patients. (**A**) The correlation of age with FEV1%Pred; (**B**) The correlation of Plasma miR-486-5p with FEV1%Pred; (**C**) The correlation of Plasma miR-106-5p with FEV1%Pred; (**D**) The correlation of Plasma SP-D mRNA with FEV1%Pred; and miR-106-5p expression; (**E**) The correlation of Plasma SP-D protein with FEV1%Pred; F. ROC curves for miR-486-5p, miR-106-5p, SP-D expression and for logistic regression model.

Age, gender, smoking history, plasma miRNA-106-5p, miRNA-486-5p, SP-D protein and SP-D mRNA expression were performed to estimate the correlation with FEV1%Pred in Tibetan COPD patients, which is the most important factor for the estimation of COPD severity. There was no significant correlation between gender, smoking history with FEV1%Pred. while age is positively correlated with FEV1%Pred (Fig. 4A). At the same time, plasma miR-106-5p and miR-486-5p were negatively correlated with FEV1%Pred, with the correlation index of − 0.528 and − 0.563, respectively (*P* < 0.05, Fig. 4B, C). Moreover, plasma SP-D protein and SP-D mRNA expression were positively correlated with FEV1%Pred, with the correlation index of 0.499 and 0.457, respectively (*P* < 0.05) (Fig. 4D, E).

ROC curves were determined for Tibetan COPD discrimination. Overall, SP-D protein level, SP-D mRNA level, miR-106-5p and miR-486-5p were all significantly discriminate (*P* < 0.05) Tibetan COPD patients from the Tibetan healthy subjects with AUCs of 0.663, 0.833, 0.869 and 0.864, respectively (Fig. 4F, Table 5). Whereas age, sex, and smoking history were not significant for Tibetan COPD discrimination. Binary logistic regression analysis of risk factors associated to Tibetan COPD was performed. Age, sex, smoking history, SP-D protein level, SP-D mRNA level, miR-106-5p, and miR-486-5p were included in the model. Age, sex, smoking history, and SP-D protein expression were not significant and, therefore, excluded from the model. Comparison of the expected and observed frequencies by the Hosmer–Lemeshow goodness-of-fit test (*P* < 0.05) and by ROC curve (AUC = 0.953; *P* < 0.05) indicated a good fit for the model. B, SE, Wald X^2^, *P*-value and Odds Ratio (O.R.) are indicated in Table 4 (Table 5).

**Table 4:** Binary logistic regression of risk factors associated to Tibetan COPD.

	B	SE	Wald X^2^	*P*	O.R. (95%CI)
miR-106-5p	− 0.681	0.244	7.789	0.005	0.506 (0.314–0.817)
miR-486-5p	− 0.791	0.281	7.913	0.005	0.454 (0.261–0.787)
SP-D mRNA	4.031	1.531	6.929	0.008	56.327 (2.800–1133.076)
Constant	1.124	1.211	0.861	0.354	3.076

**Table 5:** Receiver operating characteristic (ROC) curve of Tibetan COPD.

	AUC	SE	95%*CI*	Cut-off	Se (%)	Sp (%)	*P*
SP-D protein	0.663	0.065	0.535, 0.790	39.351	40.0	94.3	0.019
SP-D mRNA	0.833	0.052	0.732, 0.935	0.9536	80.0	85.7	0.000
miR-106-5p	0.869	0.043	0.784, 0.954	3.571	91.4	71.4	0.000
miR-486-5p	0.864	0.044	0.777, 0.952	4.707	100	62.9	0.000
miR-106-5p, miR-486-5p, SP-D mRNA	0.953	0.022	0.909, 0.995	0.661	85.7	91.4	0.000

AUC: Area under the curve; 95% CI: 95% confidence interval; Se: Sensitivity; Sp: Specificity;

## Discussion

This is the first study to investigate a specific differentially expressed miRNA profile and surfactant protein between Tibetan healthy people and Tibetan COPD patients. The present study aimed to identify the involvement of miRNAs and surfactant protein in the pathophysiology of COPD and to explore their effects with significant alteration on Tibetan COPD in vitro.

The pathogenesis of COPD is very complicated, which is affected by the combination of environmental and genetic factors^16^. Smoking, passive smoking, education level, occupational exposure, and seasonal climate all influence the incidence of COPD. Compared with the Han population, the environmental exposure and genetic background of Tibetan residents are very different, and the disease spectrum of Tibetans and Hans living in plateau areas is different, suggesting that genetic factors may be involved in the susceptibility of different races to diseases. A variable number of differentially expressed miRNAs have been reported among individuals affected by COPD or asthma in comparison with healthy individuals in several studies^17,18^. but few studies are focus on Tibetan people. In this study, we found that there were 210 differentially expressed miRNAs between Tibetan COPD patients and Tibetan healthy people, with 124 downregulated miRNAs and 86 upregulated miRNAs. Consistent with miRNAs profile, expression of miR-106b-5p and miR-486-5p were validated by qRT-PCR. We identified that miR-106b-5p and miR-486-5p expression were significant higher in Tibetan COPD patients than Tibetan healthy people.

Functional analysis of predicted gene targets for differentially expressed miRNAs revealed that these predicted target gene mainly influenced guanyl-nucleotide exchange factor activity, cell morphogenesis and the positive regulation of GTPase activity. These miRNAs are mainly enriched in signaling by NGF, Axon guidance, developmental biology, ubiquitin mediated proteolysis, and PDGF signaling pathway. Among them, developmental biology was enriched the most target genes. KEGG enrichment of predicted target gene showed pulmonary arterial hypertension was the 14th enriched disease which is the main complication of COPD. OMIM enrichment showed lung cancer was the 1st enriched disease which consistent with that COPD patients at higher risk of developing lung cancer^13^. COPD-related biomarkers were the 30th enriched disease by NHGRI GWAS Catalog enrichment analyses. Although accurate functional studies should be performed to validate this, we suggest that targeting NGF or PDGF signaling pathway could be as novel therapeutic approaches for treating COPD.

Even though pulmonary is the main expression site of surfactant proteins (SP), it has been localized to glandular system^19^, reproductive tract^20^, urinary tract^21^, and in the cardiovascular system^22^. The protein and mRNA expression of plasma SP-D in Tibetan COPD patients have not been reported. A previous study showed that pulmonary SP-D levels were lower than healthy subjects^23^. In addition, extracellular vesicles (ECVs) are secreted cell-derived membrane particles involved in intercellular signaling and cell–cell communication, which exist wildly in blood. This study showed that the plasma mRNA expression of SP-D in Tibetan COPD is lower than healthy people. Lots of studies had shown that the protein levels of SP-D in COPD plasma were increased, and correlated with the severity of COPD^24,25^. However, this study showed that plasma SP-D protein level were decreased in Tibetan COPD patients compared with healthy Tibetan subjects. This result may be due to the unique adaptability of Tibetan population under hypoxia. SP-D usually shows anti-inflammatory properties and dampens local inflammation in the vessel. However, SP-D can also exert a pro-inflammatory role by stimulating blood monocytes to secrete tumor necrosis-factor α. In vivo studies SP-D plays a proatherogenic role, with SP-D knockout mice having smaller atherosclerotic plaque areas^26^. Chronic pulmonary heart disease is one of the major complications of COPD. therefore, decreased plasma SP-D protein level in Tibetan COPD patients may have a protective effect against the risk of cardiovascular disease in COPD.

COPD is the fourth leading cause of mortality, and is predicted to be the third leading cause of death worldwide by 2020^27^. It is known that low lung function is associated with high mortality risk, due to COPD particularly. Therefore, it is of very importance to study genetic aspects which would increase the susceptibility of COPD and lung function decline. In this study, we found that miR-486-5p and miR-106-5p were all negatively correlated with FEV1%Pred. Moreover, the protein and mRNA expressions of plasma SP-D were positively correlated with FEV1%Pred, and maybe as biomarkers to reflect the severity of Tibetan COPD. Therefore, plasma miR-486-5p, miR-106-5p, the mRNA and protein expression of SP-D may as biomarkers to the estimation of Tibetan COPD severity.

Binary logistic regression analysis showed plasma miR-106-5p, miR-486-5p and SP-D mRNA level were the risk factors of Tibetan COPD. ROC curves results showed miR-106-5p, miR-486-5p, SP-D mRNA level and SP-D protein level may all discriminate Tibetan COPD patients from the Tibetan healthy subjects, while miR-106-5p is the best model. In contrast, an integrated logistic regression model (combination of plasma miR-106-5p, miR-486-5p and SP-D mRNA level) was better than miR-106-5p model and showed an adequate discriminatory potential to assist the diagnosis of Tibetan COPD.

In future work, more cases are needed to further identify the above results, and functional studies also should be performed for the therapy of COPD.

## Conclusion

The present study is the first to show significant differential expressed miRNAs between Tibetan COPD and Tibetan healthy subjects. In addition, we also measured the plasma protein and mRNA expression of SP-D in Tibetan COPD and healthy people for the first time. Moreover, our results have shown that age, plasma miR-106-5p, miR-486-5p, SP-D mRNA level and SP-D protein level were all correlated with FEV1%Pred, and may as the risk factors of Tibetan COPD. The combination of plasma miR-106-5p, miR-486-5p and SP-D mRNA expression maybe the best model to assist the diagnosis of Tibetan COPD. Thus, suggesting that different pathophysiological mechanisms may underlie COPD and therefore, different diagnosis and treatment approaches should be considered for Tibetan COPD.
